# Intestinal barrier function of Atlantic salmon (*Salmo salar *L.) post smolts is reduced by common sea cage environments and suggested as a possible physiological welfare indicator

**DOI:** 10.1186/1472-6793-10-22

**Published:** 2010-11-09

**Authors:** Henrik Sundh, Bjørn Olav Kvamme, Frode Fridell, Rolf Erik Olsen, Tim Ellis, Geir Lasse Taranger, Kristina Sundell

**Affiliations:** 1 Fish Endocrinology Laboratory, Department of Zoology/Zoophysiology, University of Gothenburg, PO Box 463, S-405 30, Gothenburg, Sweden; 2 Institute of Marine Research, P.O. Box 1870 Nordnes, 5817 Bergen, Norway; 3 Institute of Marine Research, N-5984 Matredal, Norway; 4 Cefas Weymouth Laboratory, Barrack Road, The Nothe, Weymouth, Dorset, DT4 8UB, UK; 5 PHARMAQ AS, P.O. Box 267 Skøyen, Oslo, Norway

## Abstract

**Background:**

Fish farmed under high intensity aquaculture conditions are subjected to unnatural environments that may cause stress. Therefore awareness of how to maintain good health and welfare of farmed fish is important. For Atlantic salmon held in sea cages, water flow, dissolved oxygen (DO) levels and temperature will fluctuate over time and the fish can at times be exposed to detrimentally low DO levels and high temperatures. This experimental study investigates primary and secondary stress responses of Atlantic salmon post smolts to long-term exposure to reduced and fluctuating DO levels and high water temperatures, mimicking situations in the sea cages. Plasma cortisol levels and cortisol release to the water were assessed as indicators of the primary stress response and intestinal barrier integrity and physiological functions as indicators of secondary responses to changes in environmental conditions.

**Results:**

Plasma cortisol levels were elevated in fish exposed to low (50% and 60% saturation) DO levels and low temperature (9°C), at days 9, 29 and 48. The intestinal barrier function, measured as electrical resistance (TER) and permeability of mannitol at the end of the experiment, were reduced at 50% DO, in both proximal and distal intestine. When low DO levels were combined with high temperature (16°C), plasma cortisol levels were elevated in the cyclic 1:5 h at 85%:50% DO group and fixed 50% DO group compared to the control (85% DO) group at day 10 but not at later time points. The intestinal barrier function was clearly disturbed in the 50% DO group; TER was reduced in both intestinal regions concomitant with increased paracellular permeability in the distal region.

**Conclusions:**

This study reveals that adverse environmental conditions (low water flow, low DO levels at low and high temperature), that can occur in sea cages, elicits primary and secondary stress responses in Atlantic salmon post smolts. The intestinal barrier function was significantly affected by prolonged hypoxic stress even when no primary stress response was observed. This suggests that intestinal barrier function is a good experimental marker for evaluation of chronic stress and that it can be a valuable tool to study the impact of various husbandry conditions on health and welfare of farmed Atlantic salmon.

## Background

The global increase in production of fish under high intensity aquaculture conditions has increased the awareness of husbandry conditions to maintain good health and welfare of farmed fish [1-4]. The concept of welfare for fish is under discussion but has been defined as the absence of suffering by some authors [5]. Fish farming results in a range of unnatural environments that may constitute a threat to the homeostasis of the fish and can be defined as stressors [6]. Thus, it is important to include stress as a central topic in the discussions on welfare and health of all intensively farmed animals including fish [7,8] as long term stress can result in pathological conditions [6,9,10]. In aquaculture, stress can be caused by a suboptimal or rapidly changing environment or by various husbandry procedures. The initial stress response includes alterations in behavioral and physiological mechanisms to cope with the new and challenging situations and is generally regarded as adaptive [11,12]. However, prolonged or repeated periods of stress as well as severe stress can result in adverse effects on various physiological systems which may have consequences for welfare and lead to compromised health and performance [6,9].

Water oxygen levels have been suggested as a key limiting factor in intensive farming of salmonids and are considered one of the most important factors affecting health and welfare [1]. In salmon aquaculture, dissolved oxygen (DO) levels in a sea cage can be highly variable both temporally and spatially and is affected by factors like stocking density, degree of stratification, water currents, position of the sea cages and daily and seasonal variations [13,14]. Tidal cycles have a strong influence on farms situated in fjords sheltered from other causes of water movement like wind, waves and prevailing currents. In these environments cyclic drops in DO level occur when the tidal current changes direction (slack water). Further, the combination of slack water and high water temperature during late summer causes the lowest and most critical DO levels within the cages [13]. Thus, during unfavourable environmental conditions these factors create a sub-optimal, stressful environment which can be detrimental for the fish health and welfare.

Similar to other vertebrates, a stress response in fish starts with the recognition of a potential threat to homeostasis by the central nervous system, which in turn activates primary stress response pathways. A key pathway is the hypothalamic-pituitary-interrenal (HPI) axis which results in elevated levels of the glucocorticoid hormone cortisol in the circulation [15]. Cortisol elicits secondary stress responses by affecting metabolism, osmoregulation, immune and barrier functions [15]. If sustained, these secondary responses will, in turn, affect whole animal performance such as growth, swimming capacity, disease resistance and reproduction, referred to as the tertiary stress responses [16].

Cortisol is a widely used indicator of stress in fish [17]. A vast amount of data show that plasma cortisol levels increase in response to a wide variety of physical, chemical, and biological stressors often found in aquaculture [18,19]. However, depending on the severity and duration of the stressor, plasma cortisol can either stay slightly elevated or return to basal levels even though the stressor is still present [9,15,19,20]. Cortisol may therefore serve as a reliable indicator for acute stress, but may be less useful as an indicator for chronic stress. Furthermore, due to its rapid release into the circulation after initiation of stress, blood sampling for cortisol measurements needs to be standardized in order to exclude possible effects of the sampling itself, which is a major obstacle when sampling large scale farming conditions. In order to avoid possible effects of sampling, the development of non-invasive methods is ongoing. In salmonids, free (unconjugated) cortisol is released through the gills, its release rate is correlated to plasma levels of cortisol which can therefore be used as a non-invasive indicator of cortisol status [21].

Stress in mammals has been demonstrated to affect gastrointestinal (GI) functions and cause for example impaired transport and disturbed barrier function which may, in turn, lead to increased permeability for macromolecules, microbial products, antigens and bacterial translocation [22-26]. These effects have mainly been attributed to endocrine stress responses through glucocorticoids [22,23]. Stress has similarly been shown to affect GI barrier integrity in fish. Acute stress in Atlantic salmon caused immediate damage to the intercellular junctional complexes and occasionally total loss of cell-cell contact [27]. Also, cellular damage was evidenced by loosening of microvilli and loss of cell content [27]. These damages were present up to 12 h after stress, whereas at later time points post stress, no or very few histological alterations could be observed [27]. Similar histopathological results were evident in acutely stressed rainbow trout (*Oncorhynchus mykiss *Walbaum) in an experiment where also an increased intestinal permeability 4 and 48 h after stress was demonstarted [28]. The permeability of the GI barrier is an important physiological feature as it will affect the ability to transport nutrients, water and salts as well as the translocation of harmful agents like pathogens. Thus, GI secondary stress responses may directly contribute to tertiary stress responses like decreases in growth and increases in susceptibility to pathogens and disease. Intestinal barrier function is therefore potentially valuable measures to assess detrimental effects of prolonged stress and welfare in aquaculture.

The objective of the present study was to determine whether long term exposure to sub-optimal water conditions occurring in sea cages, replicated in controlled large-scale laboratory tanks, can affect intestinal barrier function of Atlantic salmon post smolts. Furthermore, the cortisol status of the fish was assessed in plasma and using a non-invasive technique to compare the two different measures as indicators of primary responses to chronic stress. The results indicate that environments often found in sea cages are stressful to the fish and elicit both primary and secondary stress responses. Fixed low DO levels reduce the intestinal barrier function as well as absorbing functions. The implications of these results and intestinal barrier function as a possible physiological welfare indicator in aquaculture are discussed.

## Methods

### Experimental setup

#### Fish

The experiments were carried out at the Matre research station, Institute of Marine Research, Norway (61° N). Atlantic salmon post smolts (Aquagen strain) with a start weight at day 0 of 390.5 ± 3.3 g, (N = 900, n = 75 per tank) in Experiment 1 and 359.5 ± 18.9 g, (mean ± SEM., N = 27, n = 9 from three random tanks) in Experiment 2 (see below) were stocked in indoor tanks. Four treatments were run in triplicate, giving a total of 12 tanks. The fish were fed twice daily with 15-25% surplus feed. Waste feed was collected 0.5 h after each meal in order to measure feed intake and appetite at tank level, and to calculate feed rations. Fish were deprived of food for 48 h before sampling. The fish were treated according to the Norwegian national legislation for laboratory animals. All fish had been vaccinated during the freshwater stage using an oil based vaccine containing formalin inactivated bacteria and virus. Morphometric and food intake data (e.g. size, growth, feed intake rates, food conversion ration) were gathered during the experiments and are reported elsewhere (Kvamme et al., in preparation); the current manuscript is restricted to physiological measures with focus on the GI tract.

##### Experiment 1: Fixed oxygen saturations at 4 levels

This experiment aimed at mimicking the overall situation often found in sea cages when water flow decreases, DO levels are reduced to around 50%, and the low water exchange rate may cause a build up of toxic metabolites. On 23 of February 2006, 3516 individuals were netted from a holding tank, transported for 5 minutes, netted again, bulk weighed and distributed to the experimental, octagonal tanks (water depth 76 cm, 6.5 m^3^). An equal density of 16.1 kg m^-3 ^and 293 individuals in each treatment tank was obtained and all tanks were supplied with 100 L min^-1 ^of SW. Mean ± SD temperature was 8.6 ± 0.1°C and salinity was 34.2 ± 0.1 throughout the experiment. The oxygen saturation of the inlet water was measured by an Oxyguard 420 probe with a Commander logger unit (Oxyguard international A/S, Denmark, http://www.oxyguard.dk), logged every hour, and showed a value of 99.7 ± 1.8% (mean ± SD). Conductivity of the inlet water was measured using an Indumax P CLS50 sensor (Endress + Hauser) and logged every 15 or 30 minute. Following an acclimation period of 13 days (day 0 = March 8^th^), four treatment oxygen regimes in triplicate tanks were set with fixed DO levels at 50%, 60%, 70% and 80% saturation. These oxygen levels were automatically regulated by adjusting inflow (range 41-137 L min^-1^) based on the outlet oxygen readings for each individual tank. The levels of carbon dioxide (CO_2_) were well below recommended safe levels (<10 mg L^-1^; [29]) in all tanks. Nevertheless, reduced water flow created a gradual increase of CO_2 _with decreasing DO levels (from 3.3 ± 0.6 mg L^-1 ^in 80% DO group to 5.2 ± 0.9 mg L^-1 ^in the 50% DO group). The same pattern was observed in ammonia which increased from 27.9 ± 1.5 mg L^-1 ^in the 80% DO group to 29.0 ± 2.3 mg L^-1 ^in the 50% DO group. No differences were observed in water pH (8.3 ± 0.1) between any groups. Further details on water quality are presented in Kvamme et. al. (in preparation). Water flow was measured by a flow-meter (Promag 50, Endress + Hauser, http://www.endress.com) and automatically regulated by a pneumatic positioner (ABB TZIM, ABB group, http://www.abb.com). Oxygen saturation of the outlet water was monitored using individual oxygen probes in each tank outlet (Oxymax W COS41, Endress + Hauser) and mean values were logged every 15 or 30 minute. Measured as means ± SD, over the treatment period, the DO saturations were 52.3 ± 5.9%, 60.9 ± 3.9%, 69.7 ± 2.6% and 79.7 ± 1.5% (equating to DO concentrations of 4.89 ± 0.59, 5.70 ± 0.36, 6.52 ± 0.26, and 7.46 ± 0.14 mg L^-1 ^respectively). The oxygen probes were calibrated in air once a week. On two occasions (Feb 13^th ^and April 4^th^) the oxygen measurements were verified using the Winkler method [30] rendering a deviation between the averaged online registrations and Winkler results with 2.1 ± 1.0% and 0.2 ± 1.1% units (mean ± SD). In order to keep similar water current speeds within the tanks, the inlet water was divided into two inlet pipes, where the first pipe created circulation in the tank with fixed water currents, while the second supplied the remaining water needed to obtain the required oxygen saturation for each tank. Water flow, temperature, conductivity and oxygen data were monitored and processed by computer software (Controlteam, Bergen, Norway). The experiment was terminated on day 48, on April 25^th^.

##### Experiment 2: Effect of fixed and cyclic DO levels at high temperature

This experiment aimed at mimicking DO levels measured in sea cages situated in fjords that are sheltered from waves, wind and strong currents. In these situations, cyclic drops in DO levels are frequently observed during slack water and further decreased DO levels are observed during high temperatures. On January 22-24^th ^2007, an average of 307 ± 11 fish were randomly distributed to each of 12 round experimental tanks (water depth = 75 cm; 5.3 m^3^), giving a stocking density of 20.8 kg m^-3^. The tanks were supplied with seawater at a salinity of 34.1 and temperature of 8.3°C. The inlet flow was set to 60 L min^-1 ^(Promag 50 flow meter, Endress + Hauser, http://www.endress.com) and regulated by GEMÜ POS 1430 positioner (Gemü, Germany, http://www.gemue.de). This water flow sustained DO levels below 50%. Oxygen concentration at tank level was continuously logged every minute (Oxyguard 420 probe, Oxyguard International, Denmark, http://www.oxyguard.dk). Target oxygen levels and oxygen cyclic variations were achieved by supplying additional oxygen through a separate inlet with hyperoxygenated water (300-400% O_2_). Throughout the acclimation period of 27 days the DO levels were kept above 85%. After the acclimation period, at day 0 (Feb 19^th^), four different oxygen treatment regimes were initiated: fixed 50% or 85% DO levels (equating to DO concentrations of 4.01 and 6.82 mg L^-1 ^respectively), or 50% or 85% DO levels in two different 6 hour cycles (4:2 h at 85:50%; 1:5 h at 85:50%). These fluctuations were selected to mimic the variations in DO levels associated with periods of slack water at tidal reversals [13,14]. The oxygen levels were automatically computer regulated by adjusting inflow of hyperoxygenated water based on the outlet oxygen readings. Salinity and temperature was logged at several positions throughout the facilities using TST 487-1A2B temperature probe and Liquisys M CLM223/253 salinity probe (Endress + Hauser). Water flow, temperature, conductivity and oxygen data were monitored and processed by computer software (SD Matre, Normatic, Nordfjordeid, Norway). The mean ± SD temperature throughout the experiment was 16.0 ± 0.1°C. The experimental period lasted for 38 days, ending on March 29^th^.

### Blood sampling

Sequential blood samplings for plasma cortisol analysis were performed in both Experiment 1 and 2 (9 fish tank^-1^). In Experiment 1, blood was sampled from all treatment groups on days 9, 29 and 48, and in Experiment 2, on days 10 and 29. In Experiment 1, blood was collected from additional fish (4 fish tank^-1^) sampled between days 41-43 from the 80% and 50% DO groups and in Experiment 2 between days 36-38 from the fixed 85% and 50% DO groups. In order to minimize the use of experimental animals, these blood samples were obtained from fish sampled for intestines (see below). Further, an experimental design did not include a time 0-sampling point. Previous studies on the Aquagen-strain have shown low and stable plasma cortisol levels in unstressed, control fish suggesting a very low or zero risk for inherent differences between the randomly assigned fish groups at the start of the present experiment. This made the exclusion of a 0-sampling point, in order to reduce experimental animals, valid. Blood sampling was performed in two steps: 1) the fish were sampled from the tank by one quick dip netting of an excess number of fish. From these, the appropriate number of fish (4 or 9) were randomly selected and anesthetised in SW containing metomidate (12.5 mg L^-1^). 2) The fish were quickly killed with a sharp blow to the head and the blood withdrawn from the caudal vein using heparinised syringe and needle. The blood was centrifuged at 13 000 g for 3 min. The plasma was transferred to new tubes and snap frozen in liquid nitrogen and stored at -80°C until further analysis.

### Water sampling

In addition to plasma cortisol measurements, water samples were collected from both experiments for measurement of water cortisol concentrations. Assessment of the release rate of unconjugated cortisol into the water is a non-invasive approach to explore the cortisol status of fish [21]. In Experiment 1, water samples were collected at 00:00 on days 0, 3, 6, 14, 29 and 48; in Experiment 2, water cortisol samples were collected at 08:00 (prior to first feeding) on days 0, 1, 3, 7, 10, 15 and 28. The water samples (1 L) were collected in polyethylene bottles from the outflow of each tank (without disturbing the fish) and also from the main inlet water and stored at -20°C until extraction.

### Plasma and water cortisol analysis

Plasma cortisol levels were measured in unextracted plasma based on a radio immunoassay (RIA) procedure described elsewhere [31] using a cortisol antibody previously validate for this method [32]. Briefly, a sheep-anti cortisol antibody (Code: S020; Lot: 1014-180182) from Guildhay Ltd. (Guildford, Surrey, U. K.) was used. Hydrocortisone-[1,2,6,7-^3^H(N)] (NET 396, NEN Life Sciences Products, USA) was used as tracer and cortisol standards were prepared from hydrocortisone (Sigma, St. Louis, USA). For determination of radioactivity in the samples a β-counter (Wallac 1409 Liquid Scintillation Counter, Turku, Finland) was used. Intra- and inter-assay coefficient of variation (CV) for cortisol was 3.9% and 5.4% respectively. The detection limit was 0.8 ng mL^-1^. Samples below the limit of detection were assigned the value of the assay detection limit. Water cortisol samples were extracted and concentrations (ng L^-1^) were determined by RIA as described elsewhere [33] and then converted to cortisol release rates (ng g^-1 ^h^-1^) using flow rate (L min^-1^) and interpolated biomass data, as previously described [21].

### Intestinal barrier integrity and permeability

In both experiments, intestinal segments for Ussing chamber analysis were sampled towards the end of the experimental period from the extreme groups in each experiment (*ie*. 80% and 50% DO in Experiment 1 and fixed 85% and 50% DO in Experiment 2). In Experiment 1, intestinal segments were sampled between days 41-43 and in Experiment 2 between days 36-38. Four fish were sampled from each tank as described above. After blood sampling the body cavity was opened laterally and mesenteries and adipose tissue were carefully removed. The intestine, from the last pyloric caeca to the anus, was removed and opened longitudinally, divided into a proximal and a distal part and thereafter washed and placed in ice-cold salmon Ringer solution [34]. The serosa was peeled off the intestinal segments to minimize diffusion distance and ensure sufficient oxygenation of the tissue, before mounted into Ussing chambers as previously described [34]. Four mL of Ringer solution was added to each side of the intestinal epithelium and the preparations were allowed a period of 60 minutes for stabilization of the electrical parameters before the start of the experiment. The intestinal area of exposure was 0.75 cm^2^. Oxygenation and stirring were ensured by an air-lift (synthetic air containing 0.3% CO_2_) on both sides of the intestinal segments. The temperature in the Ussing chambers was maintained at the tank water temperature using water-filled cooling mantles. The electrical parameters; transepithelial resistance (TER), short-circuit current (SCC) and transepithelial potential (TEP) were measured every 5 min throughout the experimental period (150 min) as a continuous monitoring of preparation viability, transporting functions and integrity. The three electrical parameters obtained give information on the physiological and barrier functions of the intestinal epithelium. In short, TER is mainly a measure of the paracellular permeability, and SCC describes the sum of active transports taking place in the apical and basolateral membranes of the enterocytes. This, together with the passive leakage of charged molecules across the epithelium is reflected in the TEP. Results for the electrical parameters were calculated as the mean TER, TEP or SCC between 130-150 min *ie*. the last 5 measuring points of the experiments. The paracellular permeability of the intestinal epithelium was also assessed as the apparent permeability (P_app_) of the hydrophilic marker ^14^C-mannitol, a well documented paracellular permeability marker [35].

After the stabilizing period of 60 minutes, the experiment started (t = 0) by renewing the Ringer solution on the serosal side. On the mucosal side, the Ringer solution was replaced with fresh Ringer solution containing ^14^C-mannitol (spec. act. 0.04 MBq mL^-1^). The electrical parameters were continuously monitored from t = 0 and for assessment of P_app_, 50 μl of the serosal Ringer was sampled at time points 10, 15, 20, 50, 80, 85 and 90 min after start of the experiment. Radioactivity was determined in a liquid scintillation counter (Beckman LS 1801; Beckman, Fullerton, CA, USA) after adding 5 ml of Optiphase High Safe II (Wallac, Turku, Finland). P_app _was calculated using Equation (1):

(1) $$\text{Papp}=\text{d}Q/\text{d}t\times 1/AC_{o}$$

where d*Q*/d*t *is the appearance rate of mannitol in the serosal compartment of the Ussing chamber, *A *is the area of intestinal surface exposed in the chamber and *C*_*o *_is the initial concentration on the mucosal side.

### Histology

Intestinal segments were analyzed for histological damage to the epithelium in the extreme groups in each experiment (*ie*. 80% and 50% DO in Experiment 1 and constant 85% and 50% DO in Experiment 2). On day 48 in Experiment 1 and day 29 in Experiment 2, respectively, 10 fish from each group were sampled and the intestinal regions were removed as described above, but the intestines were not opened longitudinally. From Experiment 1 the proximal region was sampled, whereas from experiment 2, both proximal and distal regions were sampled. The sections were fixed in McDowell's fixative [36], stored in closed containers at 6°C in darkness until further processing. For histological preparation, the segments were washed in phosphate buffer (300-320 mOsm, pH 7.4), post-fixed for 1.5 h in osmium tetroxide, rewashed in phosphate buffer and stained *en bloc *in 2% aqueous uranyl acetate. After dehydration in a graded series of ethanol concentrations, the samples were embedded in Epon/Araldite via propylene oxide. Semithin (1.0 μm) sections were stained with 2% toluidine blue and examined under a Leica DMLB light microscope (LM) at 400 × and 630 × magnification. Images were acquired by a Leica DC 300 digital camera and further processed using Olympus Cell a-d ™ software.

### Statistics

All data were analysed using 2-factorial analysis of variance (ANOVA) in a general linear model (GLM). Normality and homogeneity of variances was tested for the residuals and predicted values in the GLM model used. Data not passing normality and/or homogeniety tests were transformed (lg_10_- transformation were sufficientin all cases). The model used for plasma cortisol levels included sampling occasion and DO treatment levels as fixed factors with tank nested within treatment. Significant effects in individual factors were subjected to Student-Newman-Keuls (SNK) post hoc test to determine differences between groups. Significant interactions, when present (*i.e*. plasma cortisol Experiment 2), was further analysed by Bonferroni corrected pair-wise comparisons. In Experiment 2, a large and variable number of plasma cortisol samples were found to be under the limit of detection of the RIA method, at each time point. These data were excluded from the GLM analysis. The number of excluded samples in each group is indicated in the results and figures. Ussing chamber data was analysed using the GLM including intestinal region and DO treatment levels as fixed factors and with tank nested within treatment. For these analyses, SPSS 18.0 (SPSS Inc., Chicago, Illinois) was used. Cortisol release rate data were analysed using a repeated measures ANOVA (Stata 9.0) to assess the effects of treatment, tank (nested within treatment) and sampling occasion. All data are expressed as means ± SEM and p < 0.05 was regarded as significant and indicated as *, p < 0.01 as ** and p < 0.001 indicated as ***.

## Results

### Plasma cortisol

#### Experiment 1

**Figure 1:**
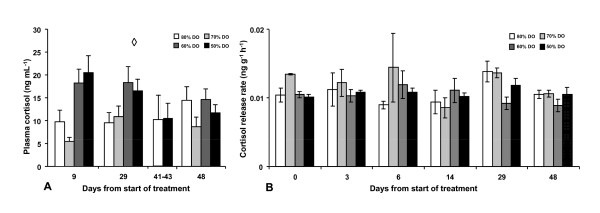
**Plasma cortisol and cortisol release rate after long term hypoxia (Experiment 1)**. This experiment aimed at mimicking an overall situation often found in sea cages when water flow decreases and DO levels are reduced to levels as low as around 50%. Decreased water exchange rate may also cause increased concentrations of toxic metabolites. Four experimental oxygen regimes in triplicate tanks were created, fixed oxygen levels at 50%, 60%, 70% and 80%, automatically regulated by adjusting inflow (range 41-137 L min^-1^) in response to oxygen consumption of the fish. Blood was sampled for plasma cortisol measurements from all treatment groups at days 9, 29 and 48 and from the 80% and 50% DO between days 41-43. Non-invasive measurement of cortisol status of the fish was conducted by measuring the cortisol release rate into the water. Plasma cortisol levels were analysed using a general linear model with sampling occasion and DO treatment levels (with tank nested within treatment) as factors. DO levels had an effect on plasma cortisol levels (A) (p < 0.001) but were not affected by time (p = 0.607) and no interaction could be observed (p = 0.128). Further, SNK post hoc test grouped the 50% and 60% DO groups in one subset and the 70% and 80% DO groups in one subset at day 9, 29 and 48. No differences could be observed in plasma cortisol between 80% and 50% DO levels in fish sampled for intestinal barrier function between days 41-43. No major differences could be observed in the cortisol release rate between treatment groups (B). All data are expressed as means ± SEM and p < 0.05. The overall effect of treatment is indicated by **◊**.

DO levels had an effect on plasma cortisol levels (p < 0.001) but were not affected by time (p = 0.607) and no interaction could be observed (p = 0.128) (Figure 1A). Further post hoc (SNK) analysis of DO treatment levels revealed two subsets of the four oxygen treatments, with 80% and 70% DO in one subset showing lower plasma cortisol values compared to the other subset, containing 60% and 50% DO (p < 0.05). Plasma cortisol levels were also analysed in blood collected from the fish sampled for Ussing chamber experiments, but only in the two extreme treatments at day 41-43, and revealed no differences between these groups.

#### Experiment 2

**Figure 2:**
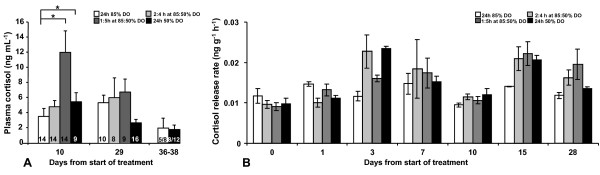
**Plasma cortisol and cortisol release rate after fixed and cyclic, low DO levels at high temperature (Experiment 2)**. This experiment aimed at mimicking DO levels measured in sea cages in fjords sheltered from waves, wind and strong currents. In these situations, cyclic drops in DO levels are frequently observed during slack water at tidal reverse and further decreased DO levels are observed during high temperatures. Four oxygen treatment regimes were initiated: fixed 50% or 85% DO levels, or 50% or 85% DO levels in two different 6 hour cycles (4:2 h at 85:50%; 1:5 h at 85:50%) at 16°C. Blood was sampled for plasma cortisol measurements from all treatment groups day 10 and 29, and from the fixed 85% and 50% DO groups between days 36-38. Cortisol levels in plasma at different time points after start of Experiment 2, assessing the impact of cyclic and fixed DO levels. Plasma cortisol samples below the limit of detection was removed and indicated in each bar (A). DO levels were not affected by sampling occasion (p = 0.572) but slightly influenced by DO treatment levels (p = 0.088). An interaction was observed (p = 0.029) and Bonferroni corrected pair-wise comparison of plasma cortisol levels revealed elevated cortisol levels in the 1:5 h at 85:50% DO group and 50% DO group compared to the 85% DO group (p = 0.002 and p = 0.012 respectively) at the first sampling after 10 days. Thereafter no differences could be observed. Overall, the cortisol release rate (B) reflected the plasma cortisol levels as there was a tendency toward lower cortisol release rate in the 85% DO group (p = 0.1). Further, plasma cortisol levels on day 29 appeared to be reflected in the cortisol release rate on day 28. All data are expressed as means ± SEM and p < 0.05 was regarded as significant and indicated as *, p < 0.01 as ** and p < 0.001 indicated as ***.

In this experiment plasma cortisol samples below limit of detection were removed in order not to violate the statistical analysis. At day 10, 14 fish from the 3 least extreme treatments (85% DO, 2:4 h at 85:50% DO, 1:5 h at 85:50% DO) and 9 fish from the 50% DO group were excluded (Figure 2A). At day 29, 10 samples were excluded from the 85% DO group, 8 samples from the 2:4 h at 85:50% DO group, 9 samples from the 1:5 h at 85:50% DO group and 16 from the 50% DO group (Figure 2A). Plasma cortisol levels were not affected by sampling occasion (p = 0.572) but slightly influenced by DO treatment levels (p = 0.088). An interaction was observed (p = 0.029) and Bonferroni corrected pair-wise comparison of plasma cortisol levels revealed elevated cortisol levels in the 1:5 h at 85:50% DO group and 50% DO group compared to the 85% DO group (p = 0.002 and p = 0.012 respectively) at the first sampling after 10 days (Figure 2A). Thereafter no differences could be observed. Moreover, all treatment groups showed the same pattern in time, *ie*. no difference between sampling occasions (p = 0.572). Also in this experiment, plasma cortisol levels were analysed in samples from the fish used for Ussing chamber experiments, but only in the the two extreme groups at day 36-38 but no differences could be revealed.

### Water cortisol

#### Experiment 1

No significant differences were observed between the different DO levels (p = 0.18) indicating that DO levels did not affected the release rate for cortisol at any of the sampling occasions (Figure 1B). Cortisol release rate was not affected by tank (p = 0.35) nor sampling day (p = 0.28).

#### Experiment 2

The cortisol release rate was not affected by tank (p = 0.97), but was affected by sampling day (p < 0.001; Figure 2B). Although the effect the different DO level regimes did not significantly affect the cortisol release rate there was a tendency (p = 0.10) towards lower release rate in the fixed 85% DO group compared to all other groups (Figure 2B).

### Intestinal barrier function

#### Experiment 1

**Figure 3:**
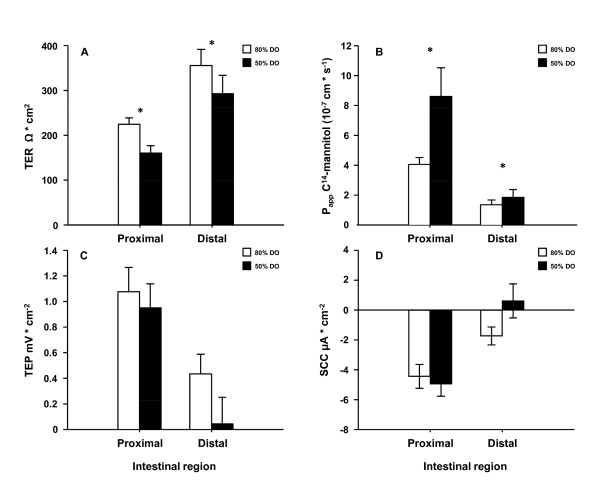
**Intestinal barrier function after long term hypoxia (Experiment 1)**. This experiment aimed at mimicking an overall situation often found in sea cages when water flow decreases and DO levels are reduced to levels as low as around 50%. Decreased water exchange rate may also cause increased concentrations of toxic metabolites. Four fish from each tank in triplicate was sampled between days 41-43, from 80% and 50% DO levels groups created by adjusting inflow (range 41-137 L min^-1^) in response to oxygen consumption of the fish. The intestine was removed and opened longitudinally, divided into a proximal and a distal part, washed in ice-cold salmon Ringer solution and mounted in Ussing chambers. The electrical parameters; transepithelial resistance (TER), short-circuit current (SCC) and transepithelial potential (TEP) were measured. TER is mainly a measure of the paracellular permeability, and SCC describes the sum of active transports. This, together with the passive leakage of charged molecules across the epithelium is reflected in the TEP. The paracellular permeability of the intestinal epithelium was also assessed as the apparent permeability (P_app_) of a well documented paracellular marker ^14^C-mannitol. Ussing chamber data was analysed using a genral linear model with intestinal region and treatment (with tank nested within treatment) as factors. Paracellular permeability was higher in both intestinal regions in the 50% DO group as indicated by decreased TER (p < 0.05) (A) and the increased P_app _for mannitol which increased in both the proximal and distal intestine (p < 0.05) (B). No major differences were observed in TEP (C) or SCC (D). All data are expressed as means ± SEM and p < 0.05 was regarded as significant and indicated as *, p < 0.01 as ** and p < 0.001 indicated as ***.

Both the proximal and distal intestines from the 50% DO treatment showed increased paracellular permeability as revealed by decreased TER (p = 0.011) (Figure 3A). In agreement, the other permeability marker P_app_, describing the diffusion rate of mannitol, was elevated in both proximal and distal intestine in the 50% DO group (p = 0.020) compared to 85% DO group (Figure 3B). No major differences were observed in TEP (Figure 3C) or SCC (Figure 3D) in the either intestinal region.

#### Experiment 2

**Figure 4:**
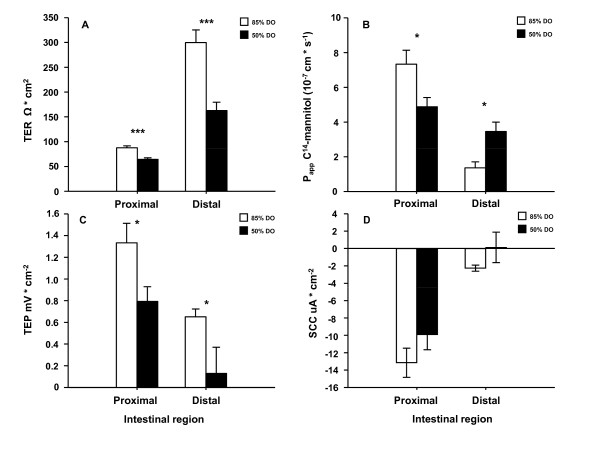
**Intestinal barrier function after long term hypoxia at high temperature (Experiment 2)**. This experiment aimed at mimicking DO levels measured in sea cages in fjords sheltered from waves, wind and strong currents. In these situations, cyclic drops in DO levels are frequently observed during slack water at tidal reverse and further decreased DO levels are observed during high temperatures. Four oxygen treatment regimes were initiated: fixed 50% or 85% DO levels, or 50% or 85% DO levels in two different 6 hour cycles (4:2 h at 85:50%; 1:5 h at 85:50%) at 16°C. Four fish per tank from fixed 85% and 50% DO groups were sampled. The intestine was removed and opened longitudinally, divided into a proximal and a distal part, washed in ice-cold salmon Ringer solution and mounted in Ussing chambers. The electrical parameters; transepithelial resistance (TER), short-circuit current (SCC) and transepithelial potential (TEP) were measured. TER is mainly a measure of the paracellular permeability, and SCC describes the sum of active transports. This, together with the passive leakage of charged molecules across the epithelium is reflected in the TEP. Ussing chamber data was analysed in a genral linear model with intestinal region and treatment (with tank nested within treatment) as factors. The paracellular permeability of the intestinal epithelium was also assessed as the apparent permeability (P_app_) of ^14^C-mannitol. TER was lower in the 50% DO group in both intestinal regions (p < 0.001) (A). An interaction between intestinal region and treatment was found for P_app _(p < 0.001), revealing a decrease in P_app _of the proximal intestine whereas there was an increase in the distal region in the 50% DO group compared to the 85% DO group (B). In both proximal and distal intestine (p < 0.05) the capability to maintain a potential difference between mucosa and serosa was reduced as shown by decreased TEP (C). No differences in SCC could be observed between treatments in neither proximal nor distal intestine (D). All data are expressed as means ± SEM and p < 0.05 was regarded as significant and indicated as *, p < 0.01 as ** and p < 0.001 indicated as ***.

Also in this experiment both intestinal regions showed disturbed paracellular integrity as shown by decreased TER (p < 0.001) (Figure 4A) and affected P_app _(Figure 4B) where an interaction between intestinal region and treatment was found (p < 0.001), revealing a decrease in P_app _of the proximal intestine whereas there was an elevation in the distal region in the 50% DO group compared to the 85% DO group (Figure 4B). Further, both intestinal regions from the 50% DO group had a reduced capability to maintain an electrochemical gradient between mucosa and serosa as shown by the decrease in TEP (p = 0.025) (Figure 4C). No significant differences were observed in SCC (Figure 4D).

### Histology

The proximal intestine was characterized by an outer layer of tightly packed granulocytes, the stratum granulosum, located on the peritoneal side of the stratum compactum. Some individual granulocytes were also located at the luminal side of this layer but the density was low. The columnar enterocytes were nicely arranged and non-vacuolarised. On occasion, small lipid droplets, stained by osmium, could be seen in the apical part of cells of the fish from Experiment 1. Mucus producing goblet cells were scattered throughout the enterocyte layer. Subjecting the fish to hypoxia tended to shorten villi height, noted in 50% of the fish examined in Experiment 1 and in 60% of the fish from Experiment 2, while the same measures for control fish were 20 and 30% respectively. No gross morphological damage to the proximal intestine could be observed in either Experiment 1 or 2. The distal intestine had a more complex structure in that it contained complex folds with substantial connective tissue and simple folds with less connective tissue. Although a distinct stratum granulosum is found in the distal intestine as well, there is a clear impression of higher number of granulocytes on the luminal side in this region compared to the proximal intestine. In the distal intestine the supranuclear cytoplasm of the enterocytes were heavily vacuolarised, ranging from very small to large vacuoles. Fish from the 50% DO group in Experiment 2 appeared to display altered appearance of the intestinal segments. When compared, reduced villi height and increased size of submucosa of the enterocyte layer was observed in 70% of the fish compared to only 30% for control fish. Although no gross morphological damage could be observed in the distal intestine, a disturbed morphology was still noticeable.

## Discussion

In this study, two experiments on Atlantic salmon post smolts were conducted in order to mimic different water currents and low oxygen conditions as well as diurnal cyclic variations in oxygen levels in combination with high temperatures, as observed during sea cage conditions [13,14]. The imitation of the complex sea cage water conditions were successfully created and maintained in controlled tank situations over several weeks. This renders, to our knowledge, the first study to achieve this and thus giving the opportunity to study physiological responses to this type of husbandry conditions.

Two main approaches were used to mimic water quality conditions that commonly occur in the sea cage environment during salmon farming [13,14]. In Experiment 1, oxygen levels were maintained at fixed levels by regulating water inflow according to the oxygen consumption of the fish, which resulted in very stable oxygen tensions over time. This reflects the sea-cage situation (oxygen levels being dependent upon the rates of consumption and replacement by water flow). Biomass and food supply were consistent across treatments and low but increases in CO_2 _and ammonium was observed whereas no changes in pH were present. The highest CO_2 _levels observed never exceeded 10 mg L^-1^. This was thus well below levels where detrimental effects of CO_2 _(> 20 mg L^-1^) for salmonids are observed [29]. Thus, most likely the results observed in Experiment 1 mainly are effects of the different experimentally set DO levels. However, it cannot be excluded that even small differences in CO_2 _and ammonium could have an impact on the physiology of the fish together with low DO levels. Therefore, although the results of Experiment 1 are discussed in relation to oxygen treatment and hypoxia in order to simplify nomenclature, other aspects of compromised water should be included in the interpretation. In contrast, in Experiment 2 the effect of oxygen was isolated from other water quality parameters: water flow was consistent between treatment groups and oxygen level was regulated by balancing uptake with addition of hyperoxic water. Treatments effects in this experiment can therefore be expected to be due solely to hypoxia. Furthermore, in Experiment 2, the temperature was set to 16°C (compared to 8.6°C in Experiment 1) as field observations indicate that the lowest DO levels in sea cages are most often observed during late summer/autumn when oxygen production from algae is low and when temperature usually is elevated as well. Thus, the two experiments have been designed to create two different types of scenarios in an attempt to mimic different environmental situations in a sea cage and are therefore expected to affect the physiology of the fish in different ways.

Environmental conditions creating a threat to homeostasis results in allostasis. This means a change in behavioural and physiological parameters to meet the environmental changes and maintain homeostasis. In allostasis, cortisol is an important regulator [9,37,38]. Salmonids are regarded as relatively intolerant to hypoxic conditions [39]. In fish, the minimum oxygen tension to provide fully saturated haemoglobin has been suggested to be 60% [29]. It can therefore be assumed that the lowest DO treatment in both experiments (50%) were stressful to the fish and thus a threat to the homeostasis. Previous work has shown that exposure to a hypoxic environment activates a cortisol response, but that there can be a rapid acclimation (habituation). In rainbow trout subjected to fixed 55% DO levels, plasma cortisol level doubled after 2 h (from ~20 ng mL^-1 ^to ~40 ng mL^-1^) but returned to basal levels after 24 h [40]. The magnitude of this plasma cortisol response to hypoxia is similar to that observed in Experiment 1, although the duration of the response differs as the elevation in plasma cortisol levels were sustained several weeks after start of treatment. Hypoxia itself can induce a primary stress response but toxic metabolites (ammonia and CO_2_) can also induce this stress response in salmonids [18,41] suggesting that a combination of these factors reveals the cortisol response in the present experiments. Even though the lack of interaction between DO levels and sampling occasion in Experiment 1 indicates that there are still a difference in plasma cortisol levels between the 50 and 60% DO level groups compared to the 70 and 80% DO level groups, the plasma cortisol levels in the lower oxygen environment have clearly decreased at the end of the experiment (day 41-43 and 48). In Experiment 2, no differences were seen between the treatment groups after day 10 and the plasma cortisol levels were low and close to basal levels [15] in all groups, as also indicated by the numerous samples excluded for being below the limit of detection for the RIA. The data on plasma cortisol levels from the two experiments taken together indicates that the fish have acclimated (habituated) to the new environments. However, it may not necessarily reflect a decreased activity of the corticosteroid system as plasma cortisol concentrations reflect the balance of the production and plasma clearance rates of the hormone from circulation. During chronic stress, increased activity including increased receptor levels and clearance rate may even render decreased plasma cortisol levels [17]. Thus, activation of the HPI axis may not be reflected by maintained increases in plasma cortisol levels during chronic stress and may therefore not be a reliable marker for chronic stress. Stocking density has been shown to reduce somatic growth although no changes, or even a decrease in plasma levels of cortisol was observed (reviewed in [1]).

The long term effect of physiological responses to stress can result in an allostatic load which is a "wear and tear" on the body and thus the cost for acclimation to sub optimal environments [42]. The allostatic load exhibited by the mimicked husbandry conditions in the present study resulted in decreased intestinal barrier functions as evident by increased intestinal permeability. TER was significantly reduced in the fixed 50% DO groups in both intestinal regions in both experiments. P_app _for mannitol increased concomitant with decreased TER in both intestinal regions in Experiment 1 and in the distal intestine in Experiment 2. Moreover, by comparing TER between the two experiments, TER appeared more reduced in Experiment 2 compared to Experiment 1 suggesting that the allostatic load from low DO levels is more severe at an elevated temperature and that hypoxia and high temperature *per se *may act as additional stressors. Increases in temperature raise the metabolism of the fish and thus oxygen demand. Concomitantly, increased temperature decreases the carrying capacity of the water to oxygen. Thus, the effects of the increased temperature, apart from being a probable stressor in itself, further ads to the hypoxic situation already created in the experimental design. Studies in mammals show that stress reduces the barrier function of the intestinal epithelium and causes increased paracellular permeability, increased uptake of macromolecules, bacterial products and antigens [24-26,43-45]. These effects are probably mediated by glucocorticoids as administration of dexametasone directly induces increased intestinal permeability in rats, an effect that was blocked by the glucocorticoid receptor antagonist RU-486 [22,23]. There is evidence that stress and corticosteroids have similar effects on intestinal integrity and permeability also in fish. Slow release cortisol implant increases the paracellular permeability of rainbow trout intestine for mannitol (K. Sundell, personal obs). Prolonged stress (hyperoxygenation and reduced water flow) in Atlantic salmon elevated plasma cortisol levels and increased paracellular permeability concomitant with increased translocation rate of pathogenic bacteria *Aeromonas salmonicida *[46]. Further, subjecting rainbow trout to an acute exhaustive stress elevated intestinal permeability up to 48 h in both the proximal and distal region [28]. Although the exact mechanism whereby hypoxia causes the detrimental changes to the intestinal barrier is unknown, it can be argued that cortisol may be an important mediator of the permeability increase.

Compared to other physiological measures of stress in fish, the increase in intestinal permeability appears to be a rather sensitive and sustainable physiologic indicator of prolonged effects of stress when other measures can be hard to interpret. Several different stressors: hyperoxygenation and reduced water flow [46], hypoxia (present study), hypoxia at high temperature (present study), IPNV infection [46], chronic feed stress [47] and high stocking density and low DO levels (Sundh et. al. in preparation) have all been shown to increase the intestinal permeability, *ie*. decrease the intestinal barrier functions after long term exposure. In agreement, although both increased cortisol levels, ultrastructural damage to intercellular junctions as well as gross morphological damage to epithelium coincided with increased permeability in acutely stressed rainbow trout, the increase in intestinal permeability was maintained and still significant at time points long after stress when ultrastructural damage and differences in plasma cortisol levels had returned to basal [28]. A similar pattern has been documented in rats where increased permeability was observed in the absence of morphological damage to intestinal tissues after stress [48]. This further indicates that the permeability increase in response to prolonged stress may not always be a result of damages to the epithelium but can also be the result of a physiological and/or immunological regulation [49-51].

In mammals, stress is discussed in relation to intestinal diseases like the inflammatory bowels diseases (IBD) [51]. These diseases are thought to result from a continuous and inappropriate activation of the mucosal immune system, driven by the enteric microflora [52]. Increased paracellular permeability, macromolecular uptake and bacterial translocation are important mediators behind the development of IBD which have major impact on the health and welfare of the diseased individuals [52,53]. The results from the current experiments suggest that prolonged stress in Atlantic salmon results in reduced barrier function similar to the observations in patients with IBD or with increased risk of developing IBD. In support, stress in Atlantic salmon has previously been shown to increases the adherence of both enteric and pathogenic bacteria to the intestinal epithelium and also cause increased translocation of live *Aeromonas salmonicida *[27,46]. The reduced barrier function will not only increase the risk for pathogenic infection, but also the delivery of antigens to the mucosal immune system with an increased immune response as a result. This may, in turn, lead to development of an intestinal inflammatory response. Intestinal inflammation, observed as increased infiltration of neutrophils, could be measured in Atlantic salmon after prolonged stress (Sundh H et. al. in preparation). All together, increased intestinal permeability in Atlantic salmon may increase the disease susceptibility and generate an IBD like physiological status with increased permeability, bacterial translocation and intestinal inflammation as common features. This should be considered as a major threat to the health and welfare of the farmed fish and thus, the intestinal permeability could be suggested to function as a physiological indicator of stress as well as welfare of Atlantic salmon.

Other aspects of the intestinal function were also affected by the experimental treatments. TEP but not SCC, was affected by the hypoxic treatment, as observed as a tendency in Experiment 1 and as a significant decrease in Experiment 2, in both proximal and distal region of the intestine. While SCC is a reflection of the overall active transports taking place in the intestinal epithelium, TEP reflects the active transport functions as well as the intestinal barriers ability to maintain an electrochemical gradient across the epithelium. Thus, the drop in TEP observed in Experiment 2 is most probably caused by a decrease in TER rather than by decreased active transport activities. The physiological consequence of a reduced TER and TEP could be impairment of nutrient uptake. The absorption of water soluble nutrients (*ie*. amino acids and glucose) are performed against their gradient mainly by use of the electrochemical gradient for Na^+ ^into the cell as created by basolaterally located Na^+^/K^+^- ATPases [54-56]. However, when TER is reduced, the paracellular leakage of Na^+ ^is increased which in turn will reduce the potential difference between the lumen and the enterocyte and reduce the gradient for Na^+ ^into the cell. This could be compensated for by increasing the activity of the Na^+^/K^+^- ATPases. However, this does not appear to occur in the present studies and therefore a reduced nutrient uptake could be an additional consequence of the stressful husbandry conditions studied. Thus, apart from increasing disease susceptibility and risk of inflammation, intestinal permeability alterations can also be a contributing factor to reduced somatic growth.

In this study, two methods for measuring cortisol status were compared, *ie*. plasma concentrations and release rate of cortisol into the water, to evaluate the sensitivity of the latter non-invasive method in a complex treatment study. The two methods gave broadly similar results. In Experiment 1 however, plasma cortisol appeared as more sensitive in distinguishing differences between treatment groups. In Experiment 2 there was a tendency towards higher cortisol release rate in fish subjected to hypoxic conditions compared to control, in line with the observations in plasma cortisol levels, which were higher in fish experiencing both cyclic and fixed low DO levels. In Experiment 1 the increased plasma cortisol on day 9 was not reflected in cortisol release rate samples taken at a close by times (day 3, 6 or 14). This disparity could be due to 1) the increased plasma cortisol on day 9 reflected a response to a non-treatment factor not present at the times the water sampling were collected 2) a lack of sensitivity in the water samples as they represent an integration over the whole tank population and over the residence time of the water within the tank, or 3) a loss of sensitivity due to normalisation of the release rate assuming a constant water flow, while in reality flow could change very rapidly as it was regulated in response to oxygen consumption of the fish. The latter reason illustrate that the non-invasive method, although having benefits [21,33], is not suitable when water flow fluctuates which is the case also in the sea cage situation. This problem in application is being addressed by searching for normalising compounds released by fish at a constant rate [21].

## Conclusions

This study demonstrates that environmental conditions with low water flow and low levels of DO, which often occurs in sea cages, can affect Atlantic salmon physiology. The environment is recognized by the fish as a threat to which they respond through activation of the HPI axis and the fish appear to be able to acclimate in terms of plasma cortisol status. However, despite acclimation in plasma cortisol, the intestinal barrier function was significantly reduced in the longer term and the disturbance was more severe at high temperatures. A reduced intestinal barrier may increases the disease susceptibility, decrease nutrient up take rendering decreased somatic growth and increases the risk of developing a chronic intestinal inflammation. Cortisol is a widely used indicator of stress in fish [17] and a vast body of evidence show that plasma cortisol levels increase in response to a wide variety of physical, chemical, and biological stressors often encountered in to aquaculture [18,19]. Although cortisol may serve as a reliable marker for acute stress, it is recognized as less suitable as an indicator of chronic stress. This, and previous studies [27,28] demonstrate that GI integrity may be compromised although plasma cortisol has returned to basal levels. Meaningful secondary responses such as the intestinal barrier function may therefore be preferable as indicators of the impact of chronic stress conditions on fish performance and can be used when evaluating the impact of husbandry conditions on the health and welfare of farmed fish.

## Authors' contributions

HS conducted the intestinal and blood samplings, all physiological experiments and analysis as well as plasma cortisol RIA analyses, statistical analysis, interpretation of results and are responsible for and corresponding author of the manuscript. BOK participated in the planning and performing of the experimental trials, blood samplings, interpretation of results and editing of the manuscript. FF participated in the planning and performing of the experimental trials, blood samplings, interpretation of results and editing of the manuscript. REO performed the histology samplings and analysis and interpretation of results and editing of the manuscript. TE performed the water samplings, analysis of water cortisol, interpretation of results and editing of the manuscript. GLT contributed with scientific ideas, in developing the experimental design, interpretation of results and editing of the manuscript. KS conceived the study, contributed with scientific conception and development of the experimental design, interpretation of results and writing of the manuscript. All authors read and approved the final manuscript.
